# Discrimination against HIV-Infected People and the Spread of HIV: Some Evidence from France

**DOI:** 10.1371/journal.pone.0000411

**Published:** 2007-05-02

**Authors:** Patrick Peretti-Watel, Bruno Spire, Yolande Obadia, Jean-Paul Moatti, for the VESPA Group

**Affiliations:** 1 Institut National de la Santé et de la Recherche Médicale (INSERM), Unit 379, “Social Sciences Applied to Medical Innovation,” Marseille, France; 2 Institut Paoli Calmettes, Marseille, France; 3 Southeastern Health Regional Observatory (ORS PACA), Marseille, France; 4 Department of Economics, University of Aix-Marseille II, France; University of Liverpool, United Kingdom

## Abstract

**Background:**

Many people living with HIV/AIDS (PLWHA) suffer from stigma and discrimination. There is an ongoing debate, however, about whether stigma, fear and discrimination actually fuel the persisting spread of HIV, or slow it down by reducing contacts between the whole population and high-risk minorities. To contribute to this debate, we analysed the relationship between perceived discrimination and unsafe sex in a large sample of French PLWHAs.

**Methodology/Principal Findings:**

In 2003, we conducted a national cross-sectional survey among a random sample of HIV-infected patients. The analysis was restricted to sexually active respondents (N = 2,136). Unsafe sex was defined as sexual intercourse without a condom with a seronegative/unknown serostatus partner during the prior 12 months. Separate analyses were performed for each transmission group (injecting drug use (IDU), homosexual contact, heterosexual contact). Overall, 24% of respondents reported experiences of discrimination in their close social environment (relatives, friends and colleagues) and 18% reported unsafe sex during the previous 12 months. Both prevalences were higher in the IDU group (32% for perceived discrimination, 23% for unsafe sex). In multivariate analyses, experience of discrimination in the close social environment was associated with an increase in unsafe sex for both PLWHAs infected through IDU and heterosexual contact (OR = 1.65 and 1.80 respectively).

**Conclusions:**

Our study clearly confirms a relationship between discrimination and unsafe sex among PLWHAs infected through either IDU or heterosexual contact. This relationship was especially strong in the heterosexual group that has become the main vector of HIV transmission in France, and who is the more likely of sexual mixing with the general population. These results seriously question the hypothesis that HIV-stigma has no effect or could even reduce the infection spread of HIV.

## Introduction

It is unfortunately a well-established fact that many people living with HIV/AIDS (PLWHA) suffer from stigma and discrimination, especially those already marginalised by gender, race and socio-economic status [1]. There is no doubt that stigma amplifies the complexities of living with HIV [2]. Moreover, there is some empirical evidence that stigma, discrimination and fear of both may contribute to an increase in HIV-related risk behaviours among both the HIV-positive [3]–[6] and HIV-negative [7]–[10] populations. Policy recommendations by international organisations in charge of the fight against the AIDS pandemic make explicit reference to this evidence by establishing a strong link between HIV prevention and access to HIV treatment and care on the one hand, and efforts targeted at reducing stigma against PLWHAs on the other hand [11]–[12]. Some social scientists have, however, recently questioned the relationship between stigma and the spread of HIV. Their argument is twofold: first they question the fact that stigma actually increases HIV-related risk behaviours within high-risk groups and those who are already infected; second they argue that even if this is the case, stigma would slow the spread of HIV infection by reducing both risk behaviours within the HIV-negative general population and sexual mixing of the whole population with those at high risk of infection [13]–[14].

The first national representative survey conducted among a large sample of HIV-infected outpatients attending French hospitals (VESPA/ANRS 2003) gave us the opportunity to analyze the relationship between PLWHAS' experience of discrimination by their social environment and their sexual risk behaviours. Such analysis may contribute to the ongoing debate about the extent to which stigma, fear and discrimination indeed fuel the persisting spread of HIV.

## Methods

### Data Collection

In 2003, the French National Agency of AIDS Research (ANRS) supported a national cross-sectional survey conducted among a random sample of 4,963 HIV-infected patients, recruited in 102 French hospital departments delivering HIV care. The methodology of this survey has been detailed elsewhere [15]. The sample was stratified on geographic location and HIV caseload. Eligible subjects were French speaking outpatients diagnosed for HIV-infection for at least 6 months, aged 18 or older, and living in France for at least 6 months. In the participating hospital units, physicians proposed the survey to a random sample of HIV-infected patients. Those who agreed to participate signed an informed consent and answered a face-to-face questionnaire administered by a trained interviewer. As patients who attend more frequently outpatient clinics were overrepresented, the sample was weighted by the inverse of patients' annual number of visits in the clinic.

The survey design has been approved by both the French Data Protection Authority (CNIL) and the National Council for Statistical Information (CNIS).

### Questionnaire

The questionnaire comprised about 400 questions, but only a subset of them have been used in the present article. Respondents were asked about their number of sexual partners during the prior 12 months and condom use with occasional and/or regular sexual partner(s). Unsafe sex was defined as reporting at least one sexual intercourse without condom with a seronegative/unknown serostatus partner during the prior 12 months.

Regarding AIDS-related stigma, participants were asked whether they have ever felt discriminated against by relatives, friends, or colleagues, due to their serostatus. Responses to these three items were collapsed into a “qualitative” binary indicator of reported discrimination in the social environment (those who have already felt discriminated against by either a relative, a friend or a colleague *versus* those who never experienced such discrimination). An alternative “quantitative” indicator was obtained by summing the three items (with a score ranging from 0 to 3). Respondents were also asked whether they have ever experienced discriminative attitudes from care providers (i.e. physicians, nurses).

The questionnaire also collected some basic medical information: transmission group (injecting drug use (IDU), homosexual contact, heterosexual contact and other), being currently treated with highly active antiretroviral therapy (HAART), CD4 cell count (documented from medical record) and symptoms of alcohol abuse during the previous 12 months (using the CAGE screening test [16]).

Finally, socio-demographic background was investigated: gender, age, educational level, being a migrant (i.e. originating from a foreign country), living in couple, and precarity of living conditions. Living conditions were considered precarious for respondents who reported financial difficulties in their household (‘It's hard to make both ends meet’, ‘we had to get into debt’) or food privation (whether or not a member of the household did not take any complete meal during a whole day due to lack of money, during the prior 4 weeks).

### Statistical Analysis

We restricted the present analysis to respondents who reported at least one sexual intercourse during the prior 12 months, and whose diagnosis of HIV infection dated back at least for one year (then unsafe sex during the previous 12 months could not have occurred before diagnosis). We used Pearson's χ^2^ to compare the reported experiences of discrimination between the three transmission groups.

Then we investigated the factors associated with unsafe sex among each transmission groups separately. Variables initially introduced in the analysis included socio-demographic background, medical information and indicators of perceived discrimination. Bivariate analysis was conducted (with Pearson's χ^2^ and Student's t-test), and dichotomous logistic models were computed for each transmission group with a stepwise selection method (entry threshold p<0.10).

## Results

### Data collected

Among the 4,963 eligible patients, 264 were not solicited because their physician considered that their physical or psychological conditions were not compatible with participation in the survey, and 1,767 patients refused to participate (2,932 participants, global response rate 59%). Patients most frequently explained their refusal by lack of time. Non-respondents were not significantly different from respondents for gender, age, viral load or CD4 lymphocyte count at time of the survey. Among participants, 2,136 reported at least one sexual intercourse during the prior 12 months and had been diagnosed HIV-infected for at least one year. Among them, 345 have been infected through injecting drug use (IDUs), 983 through homosexual contacts, and 740 through heterosexual contacts.

### Reported experiences of discrimination

Among responding PLWHAs, 12% reported experiences of discrimination from relatives (18% in the IDU group, 13% in the homosexual group, 9% in the heterosexual group, p<0.001); 12% have already felt discriminated against by a friend (16%, 12%, 11% respectively in each transmission group, p<0.04); and 7% by a colleague in their working environment (9%, 8%, 5% respectively, p<0.03). Overall, 24% of respondents reported experiences of discrimination in their close social environment with a higher prevalence in the IDU group (32%) than in the two other groups (25% and 20% respectively; p<0.001 when comparing the three groups simultaneously, p = 0.02 when comparing only the homosexual and heterosexual groups). Moreover, 27% of respondents reported attitudes of discrimination during interactions with health care providers (41% in the IDU group, 26% in the homosexual group, 21% in the heterosexual group, p<0.001).

### Factors associated with unsafe sex

Among responding PLWHAs, 18% reported unsafe sex during the previous 12 months (23% in the IDU group, 15% in the homosexual group, 19% in the heterosexual group, p<0.01) (see Table 1). In the multivariate analyses performed for each transmission group, CD4 cell count at time of the survey and perceived discrimination by care providers were never selected as significant predictors of unsafe sex.

**Table 1:** Factors associated with unsafe sex among French people living with HIV/AIDS, according to transmission group, VESPA-ANRS survey (n = 2,136, 2003).

	Intravenous drug use (n = 345)	Homosexual contact (n = 983)	Heterosexual contact, other (n = 740)	Intravenous drug use (n = 345)	Homosexual contact (n = 983)	Heterosexual contact, other (n = 740)
	*Unsafe sex vs. safe sex, row %, mean value (p value)*			*multivariate odds ratios [CI 90%]*		
Gender:						
-men (n = 1,605) (ref.)	17% vs 83%	15% vs 85%	17% vs 83%	−1-	____	NS
-women (n = 531)	37% vs 63% (0.00)	____	21% vs 79% (0.14)	2.60 [1.64;4.12]		
Being a migrant:						
-no (n = 1,719) (ref.)	22% vs 78%	16% vs 84%	21% vs 79%	NS	NS	−1-
-yes (n = 417)	26% vs 74% (0.61)	14% vs 86% (0.65)	16% vs 84% (0.07)			0.63 [0.45;0.89]
Educational level:						
-primary school (n = 107) (ref.)	15% vs 85%	23% vs 77%	18% vs 82%	NS	−1-	NS
-high school (n = 1,341)	21% vs 79%	16% vs 84%	21% vs 79%		0.52 [0.24;1.13]	
-university (n = 688)	31% vs 69% (0.24)	14% vs 86% (0.40)	16% vs 84% (0.22)		0.41 [0.19;0.90]	
Precarity of living conditions:						
-no (n = 1,504) (ref.)	20% vs 80%	16% vs 84%	17% vs 83%	−1-	NS	−1-
-yes (n = 631)	26% vs 74% (0.18)	15% vs 85% (0.87)	22% vs 78% (0.09)	1.62 [1.03;2.53]		1.62 [1.16;2.26]
Living in couple:						
-no (n = 634) (ref.)	14% vs 86%	13% vs 87%	13% vs 87%	−1-	−1-	−1-
-yes (n = 1,501)	26% vs 74% (0.01)	17% vs 83% (0.15)	21% vs 79% (0.01)	2.11 [1.24;3.60]	1.69 [1.20;2.37]	2.47 [1.61;3.79]
Number of sexual partners (prior 12 months):	1.6 vs 2.3 (0.06)	16.4 vs 9.0 (0.00)	1.8 vs 1.8 (0.99)	NS	1.02 [1.01;1.03]	NS
Alcohol abuse:						
-CAGE score<2 (n = 1,852) (ref.)	20% vs 80%	15% vs 85%	18% vs 82%	−1-	NS	NS
-CAGE score≥2 (n = 283)	31% vs 69% (0.03)	20% vs 80% (0.17)	27% vs 73% (0.07)	1.78 [1.10;2.88]		
Currently treated with HAART:						
-no (n = 328) (ref.)	29% vs 71%	17% vs 83%	33% vs 67%	NS	NS	−1-
-yes (n = 1,808)	22% vs 78% (0.28)	15% vs 85% (0.55)	17% vs 83% (0.00)			0.38 [0.26;0.54]
Discrimination in the social environment:						
-no (n = 1,624) (ref.)	18% vs 82%	16% vs 84%	17% vs 83%	−1-	NS	−1-
-yes (n = 511)	31% vs 69% (0.01)	14% vs 86% (0.48)	28% vs 72% (0.00)	1.65 [1.05;2.58]		1.80 [1.27;2.54]

Reading example: among people infected through intravenous drug use, 17% of men reported unsafe sex during the previous 12 months, *versus* 37% among women.

*p*-value for the Pearson's χ^2^ for categorical row variables (and the Student's t for number of sexual partner).

CI 90%: confidence interval, p = 0.90.

NS: variable not selected by the stepwise selection procedure (entry threshold p = 0.10).

Table 1 shows that living in couple was the only significant predictor of unsafe sex that was found in all three transmission groups by multivariate analysis. Precarity of living conditions was significantly associated with unsafe sex in both groups of PLWHAs infected through IDU and heterosexual contact. In addition, among those infected through IDU, being a woman and alcohol abuse significantly increased the likelihood of HIV-related risky sexual behaviour whereas among those infected through heterosexual contact, being a migrant and being currently HAART-treated decreased it. Regarding the subsample of respondents infected through homosexual contact, the probability to report unsafe sex decreased with the educational level, but it increased with the number of sexual partners during the prior 12 months (OR = 1.02 for each additional partner).

Moreover, results of multivariate analysis presented in Table 1 confirm the hypothesis that experience of discrimination in the close social environment is associated with an increase in unsafe sex for both PLWHAs infected through IDU and heterosexual contact. However, such relationship was not found in the case of the group infected through homosexual contacts. Results presented in Table 1 refer to the “qualititative” indicator of perceived discrimination, but identical results were obtained when alternatively using the “quantitative” indicator (estimated odds ratio per additional experience of discrimination: 1.31 among the IDU group, 1.37 among the heterosexual group).

## Discussion

Since the advent of the HIV epidemic, French public health authorities have adopted policies explicitly linking HIV prevention, free of charge access to care for all those who are HIV-positive and protection of PLWHAs against stigma and discrimination [17]. Legal measures have been introduced to protect patients with chronic diseases, including HIV/AIDS, from discrimination on the labour and insurance markets [18]. Large scale media campaigns regularly denounce discriminatory attitudes against PLWHAs and try to promote social solidarity toward them among the general population [19]. In spite of these continuous efforts, 24% of respondents in a large national random sample of French HIV-positive patients reported experiences of discrimination in their close social environment, and 27% already felt rejected by some care providers, with higher prevalences among those infected through IDU when compared to the other transmission groups. Moreover, 18% of responding PLWHAs reported unsafe sex during the prior 12 months, with again a higher prevalence in the IDU group. Regarding the relationship between perceived discrimination and unsafe sex, we found contrasted results across transmission groups. Although HIV-positive homosexual men usually experience a double stigma due to both social prejudices against their sexual orientation and their serostatus [20]–[21], this experience did not seem to interfere with their sexual practices. By contrast, those PLWHAs who had been infected through IDU reported both the highest levels of discrimination and of unsafe sex and the two phenomena remained clearly related even after multivariate adjustment. The case of PLWHAs who had been infected through heterosexual contacts is of special interest. Although they have less occasions to feel discriminated, the relationship between perceived discrimination and unsafe sex was especially strong among this heterosexual transmission group, whose members are probably the more likely to engage in ‘sexual mixing’ with the general population.

### Limitations of the present study

Before discussing our results, we must acknowledge several limitations in the present study. First, 41% of solicited patients refused to participate. Such rate of non-response is understandable given the length of the questionnaire (40 minutes for completion on average). However, we collected several characteristics of non-respondents, who were not different from respondents for several key variables (gender, age, viral load, CD4 cell count). It should also be noted that the VESPA survey was only representative of PLWHAs who are aware of their serostatus and who benefit from care in French hospitals: other groups who may contribute to the epidemiological transmission of HIV include those who have not yet been diagnosed for their HIV infection [22] and those who know their serostatus but do not seek for regular care, although this latter group is known to be of a very limited size due to the absence of economic barriers in access to HIV care in France [23]. Secondly, 99% of participants were considered “sincere” in their answers by interviewers, but we cannot exclude that some social desirability bias may have influenced self-reports of some respondents about their sexual behaviours. Answering questions dealing with unsafe sex can be intimidating in a hospital setting although the fact that data was collected outside the context of clinical interactions with prescribing physicians by trained interviewers who were totally independent from the medical staff has certainly helped to minimize such bias. Thirdly, our questionnaire did not address an important aspect of sexuality, namely the choice of partners and how perceived stigma may influence it. Finally, the present study has been conducted in France, and its results should not be generalized to other sociocultural contexts without caution. However, we believe that the impact of stigma on risk behaviours among PLWHAs may be even worse in other contexts, and especially in countries from Asia, Africa and Eastern Europe with high prevalence rates [24]–[26].

### Unsafe sex and social vulnerability

Since the advent of HAART, concerns have been raised that improvements in treatment may result in an increase of unsafe sex among PLWHAs [27]. In our study, we found no relationship between being HAART-treated and HIV-related risk behaviours in the IDU and homosexual transmission groups and even a decreased likelihood of reporting unsafe sex among those who were HAART-treated in the heterosexual transmission group. This result is in line with a meta-analysis of previous studies which concluded that HIV-positive patients receiving HAART did not exhibit increased sexual risk behavior, even when therapy achieved an undetectable viral load [28]. Previous studies have also found that alcohol use or abuse is predictive of unsafe sex among PLWHAs [29]–[30], but our results suggest that it may be specifically the case among those who already have a history of substance abuse (i.e. people infected through IDU). More generally, beyond vulnerability to substance abuse, economic vulnerability (measured by precarious living conditions) and relational vulnerability (measured by perceived discrimination from the close social environment) were significantly related to unsafe sex among both the IDU and heterosexual groups, whose living conditions are poorer than those of the homosexual group, at least in the French context [4]. Thus we should consider that stigma and discrimination are one aspect of the situations of social vulnerability faced by PLWHAs, and that the relationship between stigma and unsafe sex is embedded in such situations.

Among the homosexual transmission group, unsafe sex was more prevalent among the less educated ones and among those who reported a greater number of sexual partners during the previous 12 months. This is consistent with previous research which found a strong correlation between sexual activity and unsafe sex in this population [31]. Living in couple was also a strong predictor of unsafe sex in the homosexual subsample, as well as among the two other transmission groups. Unprotected sex among regular partners has been shown to account for a significant proportion of new HIV infections among homosexual men [32]. In a previous analysis of the subsample of homosexual men living with HIV included in theVESPA study, it was found that unprotected sex was three times more likely in seroconcordant than in serodiscordant relationships [33]. Unprotected sex has a symbolic value within a regular relationship, including those involved in serodiscordant couples: condoms may be viewed by regular partners as a symbol of distrust, dirtiness, sleaziness [34]–[35], while unprotected sex helps defining a relationship as ‘true love’, contrasting it with sexual adventures, and receiving one's partner's semen gives the feeling of ‘having him completely’ [36]–[37]. Of course, this does not mean that unsafe sex is always a “free” deliberate choice for both partners. In the present study, HIV-infected women were more likely to report unsafe sex in the IDU transmission group; according to a previous analysis of the VESPA survey among heterosexual PLWHAs, men were far more likely than women to justify unsafe sex with steady partner by their own dislike of condom use [4]. These results remind us that unsafe sex is the outcome of a social interaction between partners with unequal power to negotiate condom use, and that HIV-infected women may have difficulties in controlling sexual intercourse with seronegative men [38]–[40].

### Stigma and the spread of HIV infection

Some authors, apparently influenced by approaches borrowed from the field of socio-biology, have recently argued that HIV-related stigma does not contribute to the spread of HIV. They even proposed the following paradox that stigma and discrimination could produce positive outcomes for public health, as it would reduce the opportunities that marginalised groups have to transmit HIV to the broader population, and would consequently slow down the spread of infection in the general population [13]–[14]. In Western countries like France, such marginalised groups are usually gays and drug injectors [41]–[42].

As in many other countries, an increased incidence of sexually transmitted diseases has been reported among homosexual men in France [43]. Epidemiological data, that were contemporary of the VESPA study, show that homo/bisexual men still accounted for 24% of the 7,000 new HIV diagnoses recorded in France in 2004, with recent infections of less than six months at time of diagnosis being the most frequent in this group [44]. We however did not find any relationship between sexual risk behaviours and experience of discrimination in the population of French HIV-positive homosexual men. This finding may thus seem to corroborate the above mentioned hypothesis that stigma does not actually play a role in the dynamics of the HIV epidemics. An alternative interpretation could rather be that HIV-positive homosexual men are more or less used to manage the consequences of discrimination in their daily lives and that, in the French context, they tend to have enough economic and social power to do so [45]. In the VESPA study, HIV-positive IDUs reported being the most discriminated against and a clear link was established in this group between perceived discrimination and HIV-related sexual risk behaviours, but epidemiological trends clearly show that HIV transmission has decreased among French IDUs [44]. These facts may seem to corroborate the hypothesis that even when discrimination impacts risks of transmission in the so-called “high-risk groups”, it does not end up in fuelling the HIV epidemic. Such interpretation would however be quite unilateral and would ignore the impressive behavioural changes observed among French IDUs, in relation with the availability of harm reduction and drug maintenance treatment that have contributed to almost stop the HIV epidemic in this specific group [45], [46].

Indeed, both the results of the VESPA study and French epidemiological data seriously question the hypothesis that HIV-stigma has no effect or could even reduce the infection spread within the general population. Heterosexual transmission has become the major contributor to the HIV epidemics in France: migrants originating from sub-Saharan Africa accounted for 27% of HIV new diagnoses in France in 2004; whereas the majority of these infections has been acquired in their country of origin before they moved to France, there is some evidence that an increasing number of HIV infections among migrants now occur after they arrived in France [47]; moreover, the proportion of new HIV diagnoses among French individuals infected through heterosexual contacts (17% of the total number of cases in 2004) has increased in recent years, particularly in women [44], and French HIV-positive heterosexual men living in a couple with children have been found to be at high risk for late testing [48]. The VESPA study clearly confirms a significant relationship between discrimination and high risk behaviours in this heterosexual group that has become the main vector of HIV transmission in France and who is the more likely of sexual mixing with the general population.

Results of the VESPA Study are in line with previous research that has suggested various pathways through which stigma can contribute to the spread of HIV. First, in other cultural contexts, stigma leads some HIV-infected mothers to opt for breast-feeding instead of formula feeding because the later one would raise suspicion about their serostatus [3]. Secondly, stigma induces delays in HIV testing and non-disclosure of seropositivity to sexual partners that could both result in further transmission of HIV [5], [6]. Thirdly, perceived stigma is also correlated to poor adherence to HAART [4], [49], and poor adherence results in the development and transmission of drug resistant strains of HIV [50].

Of course, the VESPA study was focused on the French population who is already HIV-positive. However, to our knowledge, there is no empirical evidence to support the hypothesis that persistence of discrimination against PLWHAs may actually decrease the likelihood of sexual mixing with those already infected in the HIV-negative population. Since the early years of the HIV epidemic, the highest frequency of negative and discriminatory attitudes toward PLWHAs has been observed in subgroups of the general population whose socio-demographic characteristics (older age) and sexual behaviours (abstinence or monogamy) did not expose them to the risk of HIV transmission [51]. More recent surveys on knowledge, attitudes, beliefs and practices in the adult heterosexual population in France have shown a trend toward decreased condom use, particularly among those with multiple sexual partners during the prior 12 months, whereas, in the same surveys, no change had occurred in the frequency of discriminatory attitudes toward PLWHAs [52]. In other countries, like the US in the 1990's, HIV infection rates have decreased while, at the same time, discriminatory attitudes had also decreased in the general population [53]–[54].

Although studies, like the VESPA one in France, document the relationship between stigma and HIV-related risk behaviours, we must recognize that undisputable evidence of a causal link between stigma and rates of HIV infection, either based on longitudinal data or cross-countries comparisons, is still lacking in the international literature. Indeed, the establishment of such evidence may be practically out of reach for epidemiologists and social scientists: cross-countries comparisons would be hazardous because AIDS-related stigma and discrimination are rooted in variable socio-cultural contexts [25], and data on infectious rates are likely to be missing or less reliable in countries were HIV-related stigma is stronger. However, proponents of the alternative hypothesis that there is no impact of discrimination against PLWHAs on the epidemiological dynamics of HIV transmission [13]–[14] should recognize that existing empirical evidence, albeit limited, does not bring any support in favour of their “provocative” point of view.
